# Lipidomic insights into the immune response and pearl formation in transplanted pearl oyster *Pinctada fucata martensii*

**DOI:** 10.3389/fimmu.2022.1018423

**Published:** 2022-10-07

**Authors:** Hailing Wu, Chuangye Yang, Ruijuan Hao, Yongshan Liao, Qingheng Wang, Yuewen Deng

**Affiliations:** ^1^ Fisheries College, Guangdong Ocean University, Zhanjiang, China; ^2^ Development and Research Center for Biological Marine Resources, Southern Marine Science and Engineering Guangdong Laboratory (Zhanjiang), Zhanjiang, China; ^3^ Guangdong Science and Innovation Center for Pearl Culture, Guangdong Ocean University, Zhanjiang, China; ^4^ Pearl Breeding and Processing Engineering Technology Research Center of Guangdong Province, Guangdong Ocean University, Zhanjiang, China; ^5^ Guangdong Provincial Key Laboratory of Aquatic Animal Disease Control and Healthy Culture, Guangdong Ocean University, Zhanjiang, China

**Keywords:** *Pinctada fucata martensii*, lipidomics, transplantation, immune response, pearl formation

## Abstract

During pearl culture, the excess immune responses may induce nucleus rejection and death of pearl oysters after transplantation. To better understand the immune response and pearl formation, lipidomic analysis was applied to investigate changes in the serum lipid profile of pearl oyster *Pinctada fucata martensii* following transplantation. In total, 296 lipid species were identified by absolute quantitation. During wound healing, the content of TG and DG initially increased and then decreased after 3 days of transplantation with no significant differences, while the level of C22:6 decreased significantly on days 1 and 3. In the early stages of transplantation, sphingosine was upregulated, whereas PC and PUFAs were downregulated in transplanted pearl oyster. PI was upregulated during pearl sac development stages. GP and LC-PUFA levels were upregulated during pearl formation stage. In order to identify enriched metabolic pathways, pathway enrichment analysis was conducted. Five metabolic pathways were found significantly enriched, namely glycosylphosphatidylinositol-anchor biosynthesis, glycerophospholipid metabolism, alpha-linolenic acid metabolism, linoleic acid metabolism and arachidonic acid metabolism. Herein, results suggested that the lipids involved in immune response, pearl sac maturation, and pearl formation in the host pearl oyster after transplantation, which might lead to an improvement in the survival rate and pearl quality of transplanted pearl oyster.

## Introduction

Pearl oyster (*Pinctada fucata martensii*) is commonly found in the equatorial zone between the Tropic of Cancer and the Tropic of Capricorn of the Indo-Pacific and western Atlantic regions, especially in China and Southeast Asia (1, 2). It is an aquatic animal well known worldwide because it can yield high-quality pearls, accounting for over 90% of pearl production from seawater (3). Pearl production involves the step of transplantation or grafting, which comprises the transplant of a small fragment (approximately 4 mm^2^) of mantle tissue obtained from a donor oyster together with the nucleus, i.e., a spherical bead of shell material, into the host oyster gonad (4, 5). As epithelial cells grow, the grafted tissue securely adheres to the pearl oyster gonad tissue, which will eventually culminate in the formation of a pearl sac (6). Typically, pearl sac development requires 1–4 weeks during which a significant amount of matrix protein is secreted and deposited by epidermal cells of the mature pearl sac (secretory epithelium), which ultimately leads to the formation of a lustrous pearl (7). Pearl production is constituted of two stages: i) the formation of pearl sac following wound healing and oyster defense response (early than one week after transplantation); and ii) pearl sac maturation and organic matrix deposition on the bare nucleus (after one week of transplantation) (7). Therefore, pearl sac development is a crucial step for successful pearl production. Nonetheless, little is known about the immune response occurring in the donor pearl oyster mantle graft and in the host pearl oyster after transplantation until pearl sac formation. Thus, investigating the immune response in the host oyster following transplantation would contribute to ameliorating the effectiveness of pearl culture systems.

With the current advancements in cost effectiveness of high-throughput sequencing technologies, the use of omics-based research, including genomics, transcriptomics, proteomics, and metabolomics, has become more frequent in different aquaculture systems. In particular, multi-omics studies have contributed to the understanding of responses related to nuclei insertion in pearl oyster (8). Furthermore, research into the mechanisms underlying the immune response following transplanted pearl oyster revealed the involvement of key genes, proteins, and metabolic pathways (9–11). In this context, metabolomics is efficient for studying the complexity of and exploring changes in different biological systems (12). As a subfield of metabolomics, lipidomics has been widely used to comprehensive study lipids within a specific biosystem (13, 14). Lipids are the central structural membrane components of organisms (15), act as an energy source (16) and precursors of secondary messengers and transcription factors (17), as well as are involved in reproduction and sexual maturation (18), immunological responses (19), environmental adaptation (20) and signaling (21). Lipidomics-based studies on bivalves have been increasing steadily, particularly when applied to investigations on reproduction (22, 23), larval development (24–26), as well as the impact of climate change (27, 28), ocean acidification (29), and host–pathogen interactions (30–32) on bivalve production.

Thus, in order to clarify the basis of the immune response and pearl formation process, high-throughput lipidomics analysis was applied to study the serum of pearl oyster transplanted from *P. f. martensii* with the aim to identify key lipids involved in pearl sac maturation and pearl formation. The results discussed herein provide information to enlarge the current understanding on the regulatory basis of the immune response and pearl formation process in pearl oyster, thus contributing for enhancing the pearl oyster survival rate and pearl quality.

## Materials and methods

### Experimental design and sample collection

Eighteen-month-old pearl oysters and with an average shell length of 60.59 ± 4.86 mm were selected in this study. Nucleus insertion was performed in pearl oysters after preoperative conditioning. After surgical implantation, hemolymph samples were collected at different sampling points, i.e., days 0, 1, 3, 7, 15, and 30. Hemolymph was collected from blood sinus in the adductor muscle of eight host pearl oysters, submitted to centrifugation for 5 min (4°C) at 3500 rpm, and the precipitates were separated for serum collection, and stored in liquid nitrogen for subsequent analysis straightaway.

### Metabolite extraction

Briefly, 60 μL of pearl oyster serum was diluted with water to a final volume of 400 μL. Subsequently, 960 μL of MTBE:methanol solution (5:1) with the internal standard (every 960 μL of solution contained 5 μL 100 μg/mL of d7-PE(15:0/18:1), 5 μL 100 μg/mL of d7-LPC(18:1) and 9 μL 100 μg/mL of d7-TG(15:0/18:1/15:0)) was incorporated to diluted serum. After homogenization for 30 s using a vortex, sonication was performed for 10 min in an ice bath, followed by centrifugation for 15 min at 3,000 rpm, 4 °C. Subsequently, 400 μL of the supernatant was collected, and the remainder part was mixed with 400 μL of MTBE, followed by vortexing, sonication and centrifugation, and then by a collection of the newly obtained supernatant (400 μL); this procedure was repeated once. Then, supernatants were pooled and vacuum-dried at 37 °C. For analysis, 200 μL of 50% methanol diluted in dichloromethane was used to reconstitute dried samples, which were then centrifuged at 13,000 rpm for 15 min at 4 °C. For LC/MS analysis, 75 μL of the final resulting supernatant was used. The control sample consisted of a mixture of equal amounts of supernatants obtained for all serum samples.

### LC-MS/MS

A 1290 Infinity series UHPLC system (Agilent Technologies, Santa Clara, CA, USA) with a Kinetex C18 column (2.1 * 100 mm, 1.7 μm; Phenomenex, Torrance, CA, USA) was used in LC-MS/MS analysis. The mobile phase A consisted of 40% water, 60% acetonitrile, and 10 mmol/L ammonium formate. The mobile phase B consisted of 10% acetonitrile and 90% isopropanol, with 50 mL of 10 mmol/L ammonium formate per liter of mixed solvent. UHPLC runs were carried out based on the following elution gradient scheme: within the initial 12 min, 40–100% of mobile phase B; 12–13.5 min, 100% of mobile phase B; 13.5–13.7 min, 100–40% of mobile phase B; and 13.7–18 min, 40% of mobile phase B. Additional parameters were: column temperature, 45 °C; auto-sampler temperature, 4 °C; injection volume, 6 μL (POS) or 6 μL (NEG), respectively.

A triple time-of-flight mass spectrometer was used to generate MS/MS spectra on an IDA. Analyst TF 1.7 software (AB Sciex, Framingham, MA, USA) was used for a continuous evaluation of full MS scans as well as for collecting and acquiring MS data and MS/MS spectra based on preselected criteria. In each cycle, the 12 precursor ions with an intensity above 100 were chosen for MS/MS using 45 eV as the collision energy (12 MS/MS events with 50 msec of accumulation time each). Electrospray ionization source conditions were as follows: gas 1, 60 psi; gas 2, 60 psi; curtain gas, 30 psi; source temperature, 600 °C; declustering potential, 100 V; ion spray voltage floating, 5000 V or -4500 V in the positive or negative mode, respectively.

### Data analysis

LipidAnalyzer (Thermo QE HFX) was employed for automated LC-MS/MS data analysis. Raw data files in.wiff format were converted to mzXML format with ‘msconvert’ in ProteoWizard (v.3.0.6150) prior to data processing in LipidAnalyzer. Peak detection was conducted on MS1 data using CentWave algorithm in XCMS. Based on MS/MS spectra, lipid identification was performed *via* an in-house lipid spectral library, self-built by Biotree Biotech Co., Ltd., (Shanghai, China), which containing 76,361 lipids and 181,300 MS/MS spectra in total. Absolute quantitation of lipids was implemented by combining information regarding peak area, SIL-IS and RF.

Data analysis was conducted following the procedure proposed by Yang et al. (33). PCA was conducted to enable sample distribution visualization and grouping. In addition, supervised OPLS-DA was performed. VIP values of the first principal components in OPLS-DA were determined for summarizing the contribution of each variable to the model. Lipids with VIP > 1 and *P* < 0.05 (obtained with Student’s t-test) were considered as DLMs. Pathway analysis was conducted based on the Kyoto Encyclopedia of Genes and Genomes (KEGG; http://www.genome.jp/kegg/) and MetaboAnalyst (http://www.metaboanalyst.ca/) databases.

## Results

### Lipidomics of transplanted pearl oyster

**Figure 1:**
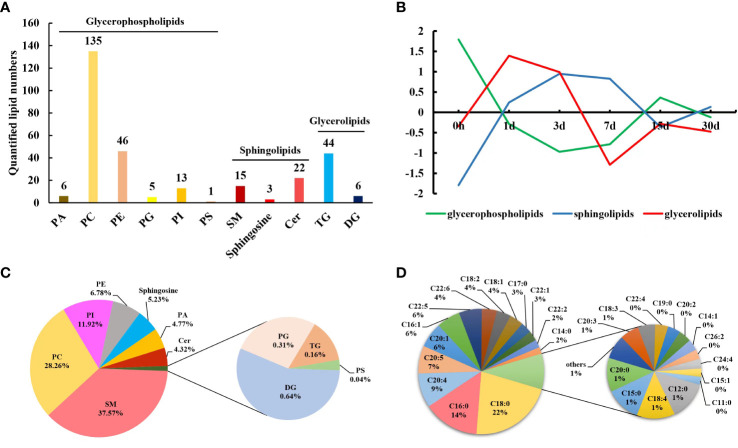
**(A)** Lipids identified in pearl oyster *P. f. martensii* after transplantation. **(B)** Changing trends of clustered lipid classes throughout different transplantation periods. **(C)** Relative proportions of different lipid subclasses in transplanted pearl oyster. **(D)** Mass percentages of membrane-esterified fatty acids in GP identified in transplanted pearl oyster.

A total of 298 lipids were identified in pearl oyster serum on different days after transplantation: 133 were in POS, and 165 were in NEG. Among these, 296 lipid species were identified by absolute quantitation and classified into three categories, namely, GP, SP, and GL, which were further subdivided into 11 subclasses, as follows: GP were divided into six subclasses, namely PC, PA, PG, PE, PS, and PI; GL were categorized into two subclasses, namely TG and DG; and SP were further divided into three subclasses, namely sphingosine, Cer and SM (**Figure 1A**). The contents of different lipids within the same subclass were considered to calculate their proportions. The proportion of SM in pearl oyster serum samples was the highest (37.57%), followed by PC, PI, PE, sphingosine, PA and Cer, accounting for 28.26%, 11.92%, 6.78%, 5.23%, 4.77%, and 4.32%, respectively (**Figure 1C**).

### Changes in lipid and fatty acids profiles in transplanted pearl oyster

To determine changes in the lipid profile in the serum of transplanted pearl oyster, the contents of lipids in each subclass discussed above were compared. The contents of GL, TG, and DG initially increased followed by a decrease, and the overall change in their contents was not significant (*P* > 0.05; **Table 1**; **Figure 1B**; **Supplementary Figure 1**). Similarly, total SP content initially increased and then decreased, with a significant difference on days 1, 3, 7, 15 and 30 compared to day 0 (*P* < 0.05; **Table 1**; **Figure 1B**; **Supplementary Figure 1**). Cer level on day 15 was significantly higher compared to that on other sampling times, whereas sphingosine content on day 3 was significantly higher than that on day 0 (*P* < 0.05; **Table 1**). Total GP content initially significantly decreased on days 1, 3 and 7, and then increased slightly (**Table 1**; **Figure 1B**; **Supplementary Figure 1**). Compared to that on other sampling points, PS content was significantly higher on day 7. PI level on day 30 was significantly higher compared to that on other sampling time points. PG content on day 3 was significantly higher than that on day 0, whereas PE level on day 15 was significantly higher than those on days 0 and 7. In contrast, PC content on day 0 was significantly higher than those on days 1, 3, and 30. Finally, PA content was significantly higher on day 3 than those on days 0 and 7 (*P* < 0.05; **Table 1**).

**Table 1:** Content of different lipid subclasses in pearl oyster serum after transplantation.

Compounds	0d	1d	3d	7d	15d	30d
Cer	0.0827 ± 0.0139b	0.0757 ± 0.0343b	0.0647 ± 0.0171b	0.0827 ± 0.0289b	0.1227 ± 0.0377a	0.0897 ± 0.0224b
SM	0.7629 ± 0.0369a	0.7709 ± 0.0438a	0.7499 ± 0.0885a	0.7618 ± 0.0459a	0.7128 ± 0.0371a	0.7500 ± 0.0410a
Sphingosine	0.0660 ± 0.0095b	0.0999 ± 0.0302ab	0.1439 ± 0.0973a	0.1121 ± 0.0215ab	0.1006 ± 0.0211ab	0.1050 ± 0.0268ab
SP	0.9166 ± 0.0283b	0.9465 ± 0.0178a	0.9586 ± 0.0071a	0.9565 ± 0.0071a	0.9362 ± 0.0162a	0.9446 ± 0.0120a
PA	0.0687 ± 0.0128b	0.1098 ± 0.0246ab	0.1292 ± 0.0572a	0.0819 ± 0.0631b	0.0918 ± 0.0320ab	0.0911 ± 0.0305ab
PC	0.6296 ± 0.0373a	0.5431 ± 0.0501b	0.5335 ± 0.0774b	0.5774 ± 0.0973ab	0.5711 ± 0.0614ab	0.5361 ± 0.0404b
PE	0.1295 ± 0.0085bc	0.1470 ± 0.0209ab	0.1325 ± 0.0213abc	0.1217 ± 0.0187c	0.1515 ± 0.0163a	0.1317 ± 0.0223abc
PG	0.0044 ± 0.0010b	0.0071 ± 0.0019ab	0.0076 ± 0.0038a	0.0063 ± 0.0041ab	0.0061 ± 0.0017ab	0.0060 ± 0.0018ab
PI	0.2402 ± 0.0274b	0.2292 ± 0.0195b	0.2213 ± 0.0257b	0.2386 ± 0.0289b	0.2274 ± 0.0182b	0.2742 ± 0.0427a
PS	0.0002 ± 0.0002b	0.0006 ± 0.0007b	0.0008 ± 0.0008b	0.0023 ± 0.0035a	0.0002 ± 0.0002b	0.0006 ± 0.0006b
GP	1.0726 ± 0.0287a	1.0368 ± 0.0177bc	1.0249 ± 0.0080c	1.0282 ± 0.00075c	1.0480 ± 0.0167b	1.0397 ± 0.0126bc
DG	0.0126 ± 0.0013a	0.0134 ± 0.0012a	0.0133 ± 0.0020a	0.0123 ± 0.0019a	0.0127 ± 0.0014a	0.0126 ± 0.0021a
TG	0.0032 ± 0.0004a	0.0033 ± 0.0004a	0.0031 ± 0.0006a	0.0030 ± 0.0004a	0.0031 ± 0.0002a	0.0031 ± 0.0005a
GL	0.0158 ± 0.0016a	0.0167 ± 0.00015a	0.0165 ± 0.0024a	0.00153 ± 0.0020a	0.0158 ± 0.0014a	0.0157 ± 0.0023a

Values are mean ± standard deviation (n = 8). Values in the same line with different small letters are significantly different (P < 0.05).

**Figure 2:**
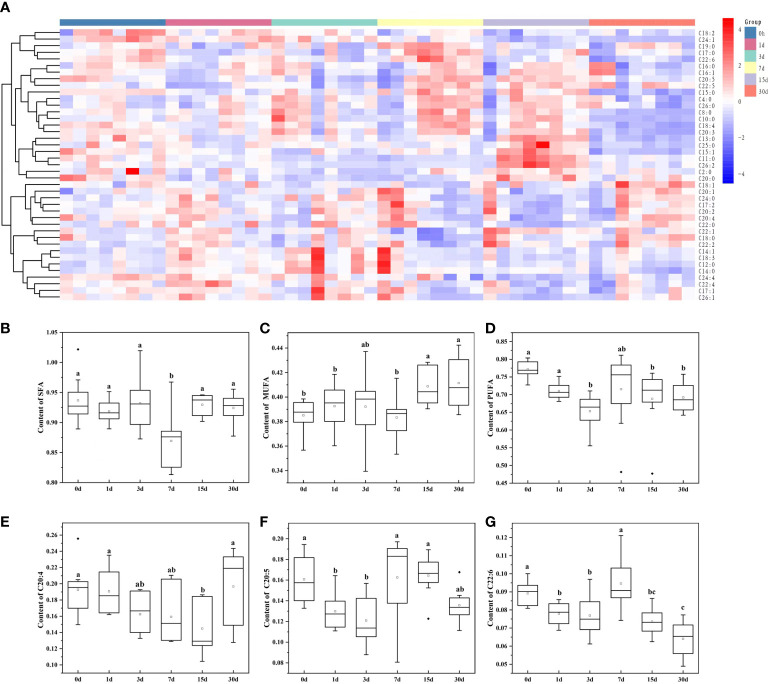
Changes in the content of membrane-esterified fatty acids in GP of transplanted pearl oyster *P. f. martensii* throughout different transplantation periods. **(A)** Heatmap analysis of 41 membrane-esterified fatty acids in GP. The relative metabolite level is depicted according to the color scale. Red and blue indicate upregulation and downregulation, respectively. **(B)** Changes in the content of membrane-esterified SFA in GP. **(C)** Changes in the content of membrane-esterified MUFA in GP. **(D)** Changes in the content of membrane-esterified PUFA in GP. **(E)** Changes in the content of membrane-esterified C20:4 in GP. **(F)** Changes in the content of membrane-esterified C20:5 in GP. **(G)** Changes in the content of membrane-esterified C22:6 in GP. Different small letters indicate significant differences between different transplantation periods (P < 0.05).

Subsequently, by analyzing the molecular structures of 206 GP, 41 fatty acids were identified in total (**Figure 2A**). Highly abundant fatty acids, including C18:0, C18:2, and C18:1, C16:0, C16:1, C20:4, C20:5, C20:1, C22:5 and C22:6, accounted for approximately 81% of total fatty acids among GP (**Figure 1D**). The levels of saturated fatty acids initially significantly decreased and then increased after transplantation, with only a low significant difference (*P* < 0.05) on day 7 (**Figure 2B**). In contrast, the levels of MUFAs were significantly higher on days 15 and 30 after transplantation (**Figure 2C**). In contrast, the levels of PUFAs were significantly lower on days 3, 15 and 30 (**Figure 2D**). An initial decrease in C20:4 and C20:5 levels was observed which was followed by a subsequent increase; specifically, a significantly low level of C20:4 was observed (*P* < 0.05) on day 15 compared to days 0, 1 and 30 (**Figure 2E**; **Supplementary Table 1**), whereas a significantly lower level of C20:5 was found (*P* < 0.05) on days 1 and 3 (**Figure 2F**; **Supplementary Table 1**). An initial decrease in C22:6 level was observed, which was followed by a subsequent increase and then a decrease, which was considered significantly lower (*P* < 0.05) on days 1, 3, 15, and 30 (**Figure 2G**; **Supplementary Table 1**).

### DLMs in transplanted pearl oyster

Findings revealed good stability and no overfitting when using the OPLS-DA model (**Supplementary Figures 2**–**4**). Therefore, it is suitable for further extension of the study. The OPLS-DA model was drawn based on normalized data, and DLMs (VIP > 1 and *P* < 0.05) in transplanted pearl oyster were determined. Up- and down-regulated DLMs in transplanted pearl oyster were determined based on five comparison groups established for pairwise comparing different transplantation periods as follows: (A) days 0 vs. 1; (B) days 1 vs. 3; (C) days 3 vs. 7; (D) days 7 vs. 15; and (E) days 15 vs. 30.

**Figure 3:**
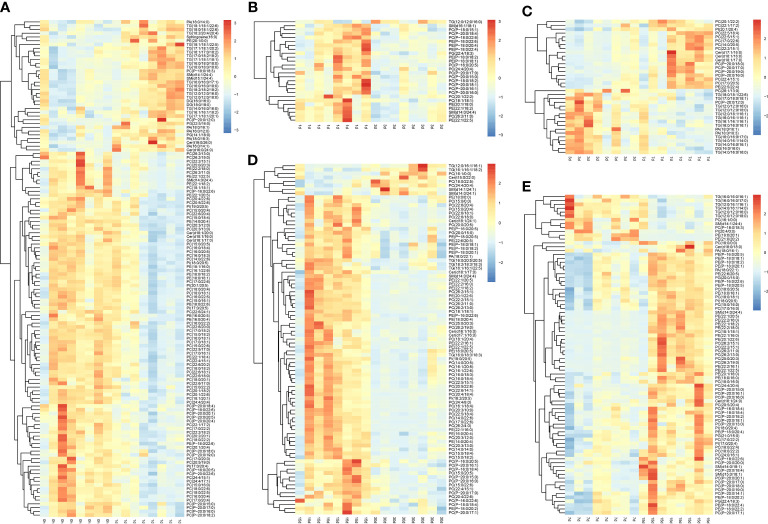
Differential lipid metabolites in transplanted pearl oyster *P. f. martensii*. The relative metabolite level is depicted according to the color scale. Red and blue indicate upregulation and downregulation, respectively. **(A)** days 0 vs. 1; **(B)** days 1 vs. 3; **(C)** days 3 vs. 7; **(D)** days 7 vs. 15; and **(E)** days 15 vs. 30.

A total of 132 DLMs (the highest number of DLMs) were identified in comparison group A, of which 35 were up-regulated and 97 were down-regulated (**Figure 3A**). GL (including TG and DG) clustered together, thus indicating a gradually increasing trend in pearl oyster after transplantation. Most GP, including PC, PE, and PI, clustered together, showing a gradually decreasing trend in pearl oyster after transplantation; in contrast, PA and PG clustered together and showed a gradual increasing trend. SP including sphingosine and several SM clustered together with an increasing trend, while Cer grouped together with mostly a decreasing trend. In comparison group B, 26 DLMs (the lowest number of DLMs) were identified, of which 25 were up-regulated and 1 were down-regulated (**Figure 3B**). Most DLMs, including PC, PE, PG, and SM, grouped together and had a decreasing trend. However, only TG (12:0/12:0/16:0) showed an increasing trend. In comparison group C, 35 DLMs were identified, of which 18 were up-regulated and 17 were down-regulated (**Figure 3C**). GL (e.g., TG and DG) clustered together and showed a gradually decreasing trend. In contrast, an increasing trend was found for SP (including Cer) and GP (i.e., PC, PE, and PI), whereas PA decreased. Considering comparison group D, 83 DLMs were identified — 71 were up-regulated and 12 down-regulated (**Figure 3D**). As expected, GL including TG were found clustered showing a decreasing trend. Similarly, GP such as PA, PC, PE, PG, and PI, as well as SP such as Cer and SM, grouped separately, and whose changing trends were towards an increase. Finally, in comparison group E 89 DLMs were identified, 8 were up-regulated and 81 down-regulated (**Figure 3E**). Most DLMs such as Cer, PA, PC, PE, PG, PI, and TG were found clustered, and whose changing trends were towards a gradual decrease. However, Cer(t15:0/22:0), PC (16:1/0:0), PC(18:0/22:5), PC(24:4/20:4), SM(d14:0/24:1), SM(d14:1/24:1), TG(12:0/16:1/16:1), and TG(16:1/16:1/18:2) grouped together with a gradually increasing trend.

### Differentially expressed lipid metabolic pathways in transplanted pearl oyster throughout different transplantation periods

**Figure 4:**
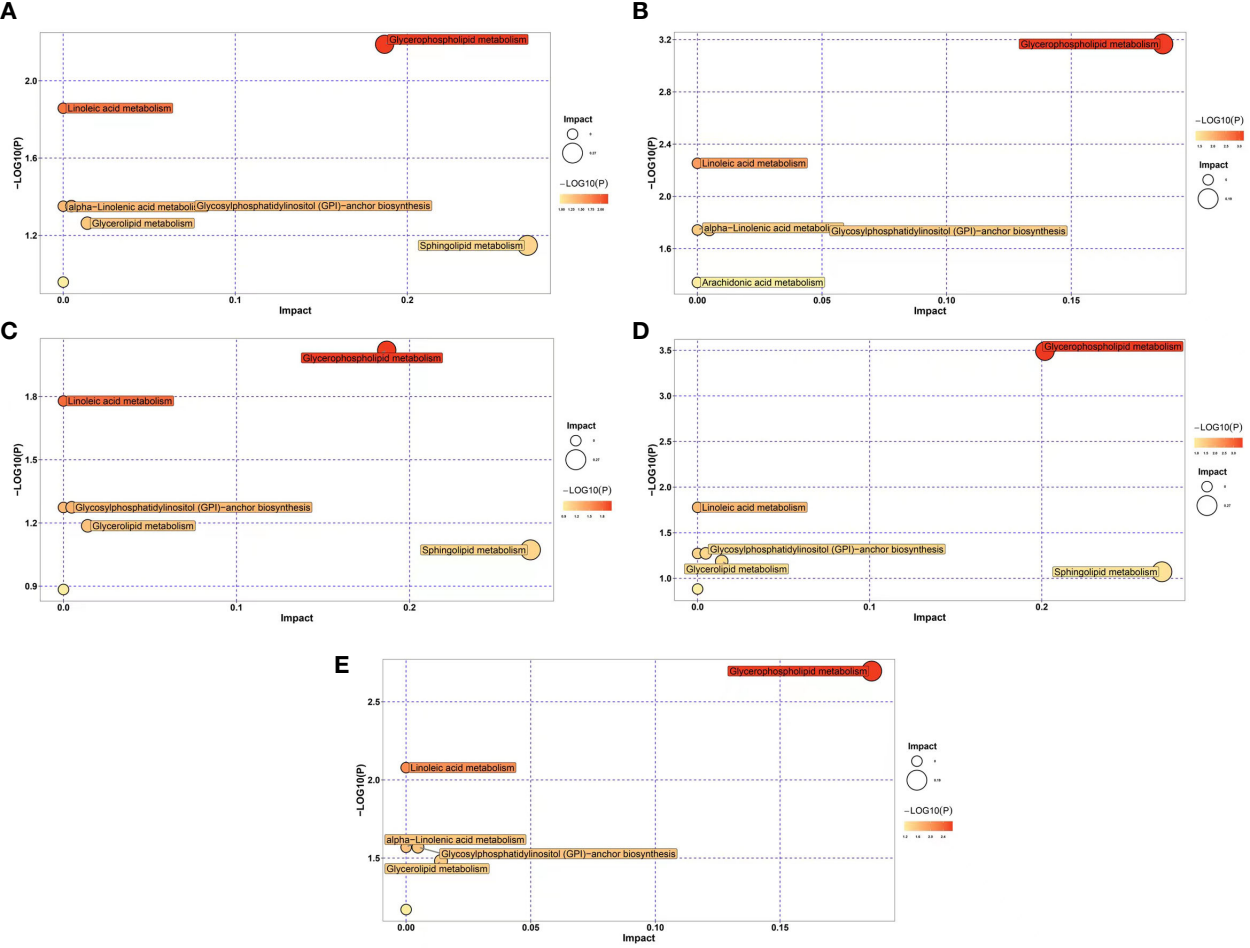
Differential expression of lipid metabolic pathways in transplanted pearl oyster *P. f. martensii* throughout different transplantation periods. The x- and y-axes represent pathway enrichment and pathway impact, respectively. Large sizes and dark colors represent major pathway enrichment and high pathway impact values, respectively. **(A)** days 0 vs. 1; **(B)** days 1 vs. 3; **(C)** days 3 vs. 7; **(D)** days 7 vs. 15; and **(E)** days 15 vs. 30.

In order to identify enriched metabolic pathways in transplanted pearl oyster, pathway enrichment analysis was conducted on DLMs based on the KEGG database. MetaboAnalyst 5.0 was used for topological analysis of enriched metabolic pathways. Five metabolic pathways were found constantly enriched in all five pairwise comparison groups discussed above (**Figures 4A–E**), namely glycosylphosphatidylinositol (GPI)-anchor biosynthesis, glycerophospholipid metabolism, alpha-linolenic acid metabolism, linoleic acid metabolism and arachidonic acid metabolism. In addition, glycerolipid metabolism was enriched in four comparison groups, i.e., **Figures 4A**, **C, D** and **E**. Similarly, sphingolipid metabolism was found enriched in comparison groups **Figures 4A**, **C**, and **4D**.

## Discussion

Lipids are vital and have very diversified roles in the physiology of living organisms, from cellular membrane building blocks to precursors of hormones and signaling molecules. The current understanding on the lipidome has been considerably enlarged by the advent of high-throughput analytical techniques (34). In pearl oyster, the wound formed after transplantation can heal or lead to cell death. In addition, other physiological responses may occur, such as foreign object rejection, disruption of the oxidative–antioxidative equilibrium, and inflammation (8). Collectively, these responses can cause nucleus rejection, failure to form a pearl sac, and ultimately lead to host pearl oyster death (35). Herein, lipidomics was applied to investigate changes in lipid profiles in the serum of pearl oyster *Pinctada fucata martensii* after transplantation.

Considering changes in lipid profiles, sphingosine levels significantly increased on day 3 of transplantation. Sphingosine controls a number of physiological processes, such as angiogenesis, immune cell trafficking, blood vessel growth, and cell survival (36), being also necessary for the synthesis of TNF-α-induced cyclooxygenase 2 and PGE2 (37). Furthermore, it has been demonstrated that intracellular sphingosine binds to and activates TRAF2 E3-ligase, leading to the Lys-63-linked polyubiquination of receptor interacting protein-1, which in turn results in the IκK complex phosphorylation and NF-κB activation, which is an important transcription factor involved in inflammatory responses (38). Moreover, the anti-inflammatory effects of sphingosine have also been described, which are related to the transformation of pro-inflammatory M1 subtype macrophage (39). In addition, sphingosine was shown to have a role in inflammation in animal models, with higher levels of sphingosine found in mice with colitis induced by dextran sulfate (40). Additionally, higher sphingosine levels were found in patients with inflammatory arthritis (41–43). Therefore, upregulated levels of sphingosine in transplanted pearl oyster may indicate a quick response to transplantation by regulating inflammatory responses.

The cholinergic system is implicated in innate immunity and inflammation in bivalve mollusks (44–47). Additionally, it has been described that the cholinergic system regulates the ability of DNA damage repair, and apoptosis in transplanted pearl oyster by affecting Ca^2+^, NF-κB, JAK/STAT, and MAPK signaling pathways (48). In the cholinergic system, acetylcholine is a key neurotransmitter in numerous physiological functions (49). After binding to a 7 nAChR, acetylcholine produced by the vague nerve suppresses NF-κB and JAK/STAT signaling pathways, which in turn lowers the inflammatory response (50). In addition, oysters treated with acetylcholine result in down-regulation of lipopolysaccharide-induced immune response by reducing the rate of hemocyte phagocytosis and apoptosis (46). Herein, PC levels were found downregulated in pearl oyster on days 1 and 3 after transplantation, which suggests that acetylcholine may participate in the regulation of immune and inflammatory responses in the early stages of grafting in pearl oysters.

Numerous PUFAs have been shown to have benefits on the health and resistance of aquatic animals. Long-chain n-3 PUFAs (DHA and EPA) have been shown to have anti-inflammatory activity that might alleviate the symptoms of chronic inflammatory diseases, allergic and trauma-enacted acute systemic inflammation (51, 52). For instance, EPA and DHA reduced interleukin-2 synthesis, mitogen-stimulation proliferation, and human natural killer cell activity in mice and human lymphocytes (52). Eicosanoids are second-class signaling molecules of the immune system connecting PUFAs to inflammatory and immunological responses (52). Eicosanoids comprise PG, thromboxanes, leukotrienes, lipoxins, and epoxyeicosatrienoic acids (53), and are synthesized from PUFAs, especially ARA and EPA. He et al. (2020) suggested a link between higher PUFA concentrations and immune system regulation in pearl oysters following grafting (54). Herein, the levels of PUFAs were downregulated on day 3 after transplantation of pearl oysters, which suggests that PUFAs may be also involved in the regulation of immune and inflammatory responses in the early stages of grafting.

Conversely, energy-intensive activities have an impact on the immunological response. PUFAs catabolism seems essential for adaptation under stress (29) in several aquatic organisms, including branchiopods (55), amphipods (56), copepods (57) and Japanese horse mackerel (58). Herein, the level of C22:6 among identified GP in transplanted pearl oyster decreased on days 1 and 3. Interestingly, significantly lower levels of PUFAs, such as C22:6, were found in marine copepods in response to environmental stress (29). Chen et al. (2014) reported that lipid synthesis capacity is increased in Pacific white shrimp *Litopenaeus vannamei* under salt stress (59). In the present study, TG and DG levels were also upregulated in pearl oyster on days 1 and 3 after transplantation, which suggests an increased rate of triacylglycerol synthesis from glyceric acid as a result of allograft-induced stress. Glyceric acid is derived from GL and carbohydrate metabolism. Decreased synthesis of glyceric acid implies that GL are mobilized as energetic supply *via* β-oxidation (60, 61). Fatty acids are degraded *via* β-oxidation, thus generating energy (12). As a result, pearl oysters may quickly deplete large quantities of glyceric acid, thus extra energy *via* fatty acid β-oxidation is likely to occur during allograft-induced stress. Previous studies have described upregulated metabolites involved in β-oxidation, which are likely to indicate lipid catabolism for energy generation during acclimation to cold stress, higher temperatures and lower pH conditions (29, 62).

Mariom et al. (2019) proposed that the crucial time for pearl sac maturation occurs during the late stages, i.e., one week to three months after grafting, during which genes involved in proliferation and differentiation are differentially expressed (7). PI is a lipid that plays a key role in the signal transduction pathway of G-protein-coupled receptors, in which extracellular signaling molecules bind to G-protein-coupled receptors on the cell surface, causing the cell to secrete, proliferate, and differentiate (63). Herein, up-regulation of PI levels in pearl oyster from days 15 to 30 after transplantation suggest that these molecules may induce cell proliferation and differentiation in different periods following transplantation. In addition, PGE 2 has been shown to mediate the effects of TGF-β, PDGF, and FGF in stimulating proliferation and differentiation (64, 65). In the present study, lower levels of C20:4 in pearl oyster from days 3 to 15 after transplantation may result from the breakdown for PGE 2 production as well as be involved in stimulating proliferation and differentiation. Hence, these lipids may induce cell proliferation and differentiation to stimulate pearl sac development.

Both living and extinct shells are known to contain fatty acids, cholesterols, phytadienes and ketones (66). Lipids represent a third of the organic matrix of calcareous biominerals. In sharp contrast, proteins constitute approximately 90% of the shell organic matrix; carbohydrates and lipids account for only 0.15–0.29% and 0.8–2.9%, respectively (67). A variety of lipids are found in the nacreous layer of *Pinctada* oysters, including cholesterols, fatty acids, and triglycerides (68). Consequently, lipids can be crucial for biomineralization and fossilization (69). According to previous studies, the regulation of aberrant bone metabolism depends chiefly on the metabolism of GP (70) and pearl oyster biomineralization activity (71). Additionally, Isa and Okazaki (1987) demonstrated *in vitro* that isolated phospholipids could bind calcium ions (72). In addition, it has been described that biomineralization-related genes involved in nacre formation were initially down-regulated 1 week and then up-regulated again after transplantation (7). Herein, GP levels were also down-regulated in pearl oyster during the first week following transplantation, and then increased. Thus, GP may be involved in pearl oyster calcification and pearl mineralization. According to a few studies in humans, LC-PUFAs can prevent bone loss, affect peak bone mass during puberty, and stimulate bone formation (33). Thus, considering that LC-PUFAs were up-regulated in pearl oyster on day 15 after transplantation (after pearl sac maturation), it can be suggested these may play a role in pearl mineralization.

## Conclusion

A total of 296 lipid species were identified by absolute quantitation, and dramatic changes were observed in serum lipid profiles of pearl oyster after transplantation. During the early stages of transplantation, sphingosine was found to be upregulated, whereas PC and PUFAs were downregulated. Interestingly, during pearl sac development stage, PI was upregulated (in between days 15 and 30). Moreover, GP levels were upregulated during pearl formation. Taken together, we speculate that these lipids might participate in the regulation of immune responses, inducing cell proliferation and differentiation and pearl mineralization in transplanted pearl oyster. It described herein enlarge the current understanding of the mechanisms underlying pearl sac maturation and pearl formation. Thus, applying the useful information discussed herein on the regulation of the immune response and pearl formation process might lead to an improvement in the survival rate and pearl quality of transplanted pearl oyster. However, more intensive investigations towards molecular mechanisms of the lipid in the immune response of pearl oyster should be performed in future studies.

## Data availability statement

The raw data supporting the conclusions of this article will be made available by the authors, without undue reservation.

## Author contributions

CY and YD designed the research. HW, CY, RH, YL, QW and YD conducted the research. HW, CY and RH analyzed data. HW, CY, RH, YL, QW and YD contributed to the final writing of the paper. All authors contributed to the article and approved the submitted version.

## Funding

This work was supported by National Natural Science Foundation of China (Grant No. 32102817), Guangdong Basic and Applied Basic Research Foundation (Grant No. 2022A1515010030), Department of Education of Guangdong Province (Grant No. 2019KQNCX043, 2020ZDZX1045 and 2021KCXTD026), and the earmarked fund for CARS-49.

## Acknowledgments

Lipidomics analysis was assisted by Biotree Biotech Co., Ltd. (Shanghai, China). We would like to thank TopEdit (http://www.topeditsci.com/) for its linguistic assistance during the preparation of this manuscript.

## Conflict of interest

The authors declare that the research was conducted in the absence of any commercial or financial relationships that could be construed as a potential conflict of interest.

## Publisher’s note

All claims expressed in this article are solely those of the authors and do not necessarily represent those of their affiliated organizations, or those of the publisher, the editors and the reviewers. Any product that may be evaluated in this article, or claim that may be made by its manufacturer, is not guaranteed or endorsed by the publisher.

## Glossary

**Table:**

PUFAs	polyunsaturated fatty acids
LC-PUFA	long-chain polyunsaturated fatty acids
MUFAs	monounsaturated fatty acids
ARA	arachidonic acid
DHA	docosahexaenoic acid
EPA	eicosapentaenoic acid
SFA	saturated fatty acid
MTBE	methyl tert-butyl ether
LC/MS	liquid chromatography-mass spectrometry
POS	Positive
NEG	negative
IDA	information-dependent basis
PCA	principle component analysis
OPLS-DA	orthogonal projections to latent structures-discriminate analysis
SIL-IS	stable isotope-labeled internal standard
RF	response factor
VIP	Variable importance in projection
DLMs	differential lipid metabolites
GP	glycerophospholipids
SP	sphingolipids
GL	glycerolipids
PC	glycerophosphatidylcholine
PA	glycerophosphatidic acid
PG	glycerophosphatidylglycerol
PE	glycerophosphatidylethanolamine
PS	glycerophosphatidylserine
PI	glycerophosphatidylinositol
TG	triacylglycerol
DG	diacylglycerol
Cer	ceramide
SM	sphingomyelin
PG	prostaglandins
PGE2	prostaglandin E2
nAChR	nicotinic acetylcholine receptor
NF-κB	nuclear factor kappa B
JAK/STAT	janus kinase/signal transducer and activator of transcription
MAPK	mitogen-activated protein kinase
TNF	tumor necrosis factor
TRAF	tumor necrosis factor receptor-associated factor
TGF-β	transforming growth factor-β
PDGF	platelet derived growth factor
FGF	fibroblast growth factor
