# Supplementary material for: Lipidomic insights into the immune response and pearl formation in transplanted pearl oyster Pinctada fucata martensii
Source: Front Immunol. 2022 Oct 7;13:1018423. doi: 10.3389/fimmu.2022.1018423 (PMC9585204; doi:10.3389/fimmu.2022.1018423)
Supplement: Supplementary file 1 [file Table_1.docx]

Supplementary Table 1 Content of different fatty acids in glycerophospholipids in pearl oyster serum after transplantation

| Compounds | 0d | 1d | 3d | 7d | 15d | 30d |
| --- | --- | --- | --- | --- | --- | --- |
| C2:0  C4:0  C8:0  C10:0  C11:0  C12:0  C13:0  C14:0  C14:1  C15:0  C15:1  C16:0  C16:1  C17:0  C17:1  C17:2  C18:0  C18:1  C18:2  C18:3  C18:4  C19:0  C20:0  C20:1  C20:2  C20:3  C20:4  C20:5  C22:0  C22:1  C22:2  C22:4  C22:5  C22:6  C24:0  C24:1  C24:4  C25:0  C26:0  C26:1  C26:2 | 0.00031±0.00026a  0.00084±0.00022ab  0.00153±0.00044a  0.00019±0.00006a  0.00634±0.00181b  0.01930 ±0.00621b  0.00408±0.00084ab  0.02851±0.00561cd  0.00456±0.00159b  0.02461±0.00072ab  0.00617±0.00086 ab  0.31001±0.02884  0.13000±0.01492  0.06245 ±0.00616ab  0.00194±0.00018  0.00118±0.00020  0.43717±0.06641a  0.07237 ±0.00707b  0.09229 ±0.02615a  0.00906±0.00148b  0.02945±0.00408a  0.00836±0.00100b  0.02888±0.00874a  0.12260 ±0.01819  0.00790±0.00078  0.01443±0.00158a  0.19266±0.03184a  0.16070±0.02373a  0.00179±0.00064a  0.04539±0.00792bc  0.03685±0.00273  0.00847±0.00098b  0.11537±0.00871abc  0.08918±0.00685a  0.00096±0.00014  0.00142±0.00042a  0.00723±0.00063a  0.00069±0.00037ab  0.00085±0.00011  0.00063±0.00015ab  0.00672±0.00229b | 0.00017±0.00011ab  0.00092±0.00022ab  0.00173±0.00067a  0.00022 ±0.00006a  0.00447±0.00077b  0.02828±0.00514ab  0.00318±0.00067c  0.03533±0.00482abc  0.00839±0.00255ab  0.02083±0.00150c  0.00520±0.00046c  0.28806±0.03205  0.11727±0.03193  0.05137±0.00302c  0.00191 ±0.00013  0.00131±0.00035  0.45408±0.04149a  0.07022±0.00777b  0.08014±0.01295ab  0.01288±0.00367ab  0.02372±0.00609a  0.00751 ±0.00195b  0.01802±0.00618bc  0.13210±0.01284  0.00914±0.00145  0.01175±0.00278a  0.19068±0.02845a  0.12978±0.01867b  0.00189±0.00082a  0.05550±0.01298ab  0.04497±0.00814  0.01048±0.00198a  0.10540 ±0.01556c  0.07798±0.00645b  0.00125±0.00044  0.00125±0.00026a  0.00695±0.00085ab  0.00057±0.00025b  0.00088±0.00013  0.00077±0.00025a  0.00530±0.00162bc | 0.00011 ±0.00006b  0.00109 ±0.00028a  0.00164±0.00119a  0.00027±0.00019a  0.00290±0.00067c  0.03664±0.0146a  0.00358±0.00062bc  0.04589±0.01556a  0.01023±0.0057a  0.02225±0.00275bc  0.00466±0.00106cd  0.30132±0.0338  0.12343±0.03798  0.05377±0.00952c  0.00192±0.00056  0.00117±0.00032  0.43566±0.06745a  0.06771±0.00384b  0.07732±0.00912ab  0.01478±0.00711a  0.02594±0.00695a  0.00772±0.00202b  0.01432±0.00233c  0.12866±0.01433  0.00849±0.00238  0.01427±0.00559a  0.16247±0.02503ab  0.12094±0.02453b  0.00185±0.00088a  0.05362 ±0.01163b  0.04073 ±0.00994  0.00882±0.00187ab  0.11079±0.01468bc  0.07693±0.01207b  0.00134±0.00041  0.00120±0.00033a  0.00684±0.00124ab  0.00057±0.00011b  0.00094 ±0.00015  0.00077 ±0.00038a  0.00394±0.00059c | 0.00018±0.00011ab  0.00112±0.00036a  0.00194±0.00143a  0.00030±0.00016a  0.00286 ±0.00087c  0.02749±0.0189ab  0.00320±0.00051c  0.04015±0.01533ab  0.00750±0.00633ab  0.02548±0.00321a  0.00533±0.00072bc  0.29747±0.04597  0.12569±0.04287  0.06757±0.00923a  0.00181±0.00046  0.00140±0.00072  0.36978±0.05899b  0.06625±0.00689b  0.07117±0.01279bc  0.01150±0.00697ab  0.02924±0.01119a  0.01050±0.00148a  0.01662±0.00471bc  0.14213±0.03055  0.00809±0.0029  0.01366±0.00566a  0.15926±0.03505ab  0.16248±0.04528a  0.00210±0.00095a  0.03322±0.01361c  0.03839±0.01296  0.00811 ±0.00182b  0.12760±0.02625ab  0.09459±0.01519a  0.00108±0.00063  0.00090±0.00026b  0.00581±0.00180bc  0.00054±0.00019b  0.00096±0.00019  0.00051±0.0004ab  0.00384±0.00061c | 0.00029±0.00007a  0.00104±0.00037a  0.00167±0.00088a  0.00029±0.00013a  0.00990±0.00342a  0.02076±0.00518b  0.00472±0.00132a  0.03304±0.00392bc  0.00547±0.00219b  0.02418±0.00400ab  0.00682±0.00119a  0.28675±0.04844  0.13068±0.04562  0.05791±0.00893bc  0.00171±0.00033  0.00101±0.00056  0.44736±0.06824a  0.07177±0.00969b  0.06021±0.01127c  0.00867±0.00314b  0.02597±0.00955a  0.00736±0.00100b  0.03024±0.00788a  0.12334±0.02248  0.00716±0.00324  0.01387±0.00524a  0.14485±0.03159b  0.16434±0.02035a  0.00105±0.00033b  0.06811±0.01316a  0.04881±0.01273  0.00753±0.00213b  0.13208±0.01577a  0.07357±0.00755bc  0.00089±0.00041  0.00053±0.00022c  0.00486±0.00051c  0.00107±0.00062a  0.00095±0.00017  0.00034±0.00018b  0.01351±0.00561a | 0.00019±0.00007ab  0.00070±0.00035b  0.00027±0.0002b  0.00005±0.00001b  0.00440±0.00081b  0.01711±0.00561b  0.00224±0.00036b  0.02228±0.00731d  0.00610±0.00264ab  0.01761±0.00167d  0.00386±0.00043d  0.27642±0.06938  0.12053±0.05788  0.05395±0.00613c  0.00169±0.00038  0.00133±0.00053  0.49432±0.05999a  0.08336±0.01221b  0.06653±0.0096bc  0.00904±0.0024b  0.01251±0.00367b  0.0080±0.002251b  0.02222 ±0.00609b  0.14147±0.02349  0.00799±0.00261  0.00552±0.00158b  0.19671±0.04778a  0.13561±0.01668ab  0.00207±0.00049a  0.05335±0.01477b  0.04484±0.01293  0.00738±0.00193b  0.13044±0.02371ab  0.06401±0.00981c  0.00131±0.00044  0.00043±0.00005c  0.00543±0.0012c  0.00024±0.0001c  0.00081±0.00016  0.00060±0.00025ab  0.0046±0.00169bc |

Values are mean ± standard deviation (n = 8). Values in the same line with different superscripts are significantly different (P < 0.05).
