# Supplementary material for: Lipidomic insights into the immune response and pearl formation in transplanted pearl oyster Pinctada fucata martensii
Source: Front Immunol. 2022 Oct 7;13:1018423. doi: 10.3389/fimmu.2022.1018423 (PMC9585204; doi:10.3389/fimmu.2022.1018423)
Supplement: Supplementary file 2 [file DataSheet_1.docx]

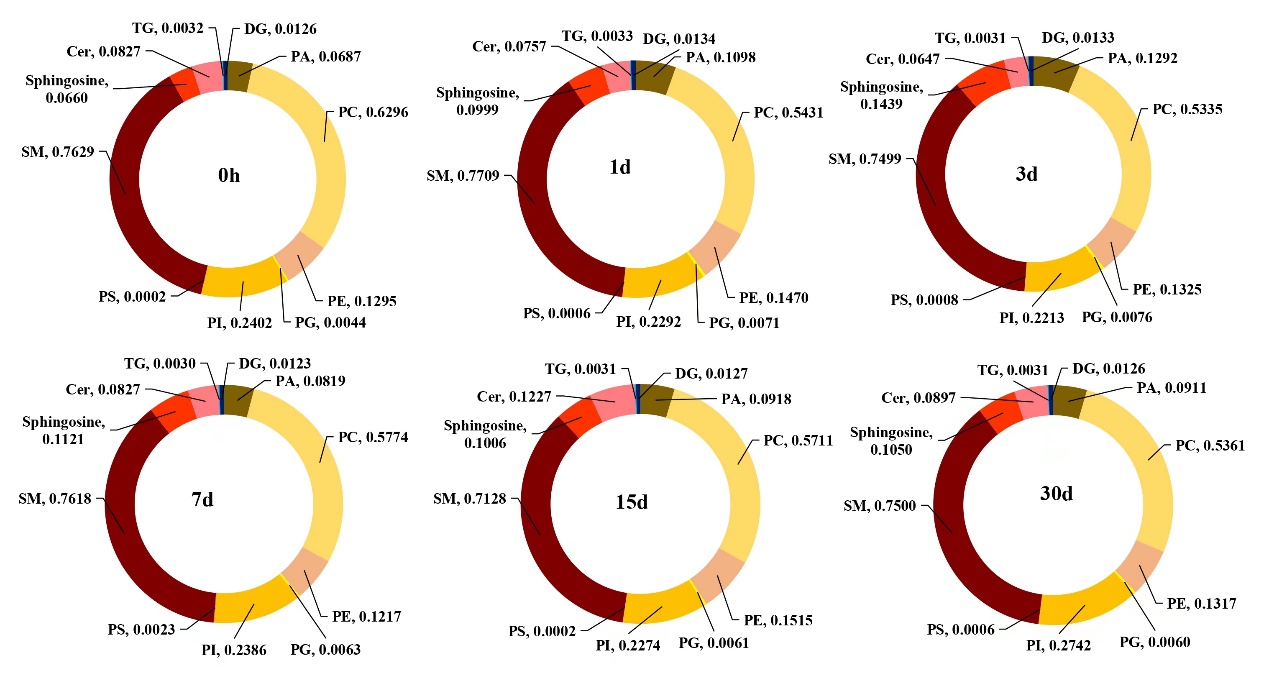


**Supplementary Fig. 1** Pie diagrams for mass percentages of quantified lipid classes in transplanted pearl oyster.


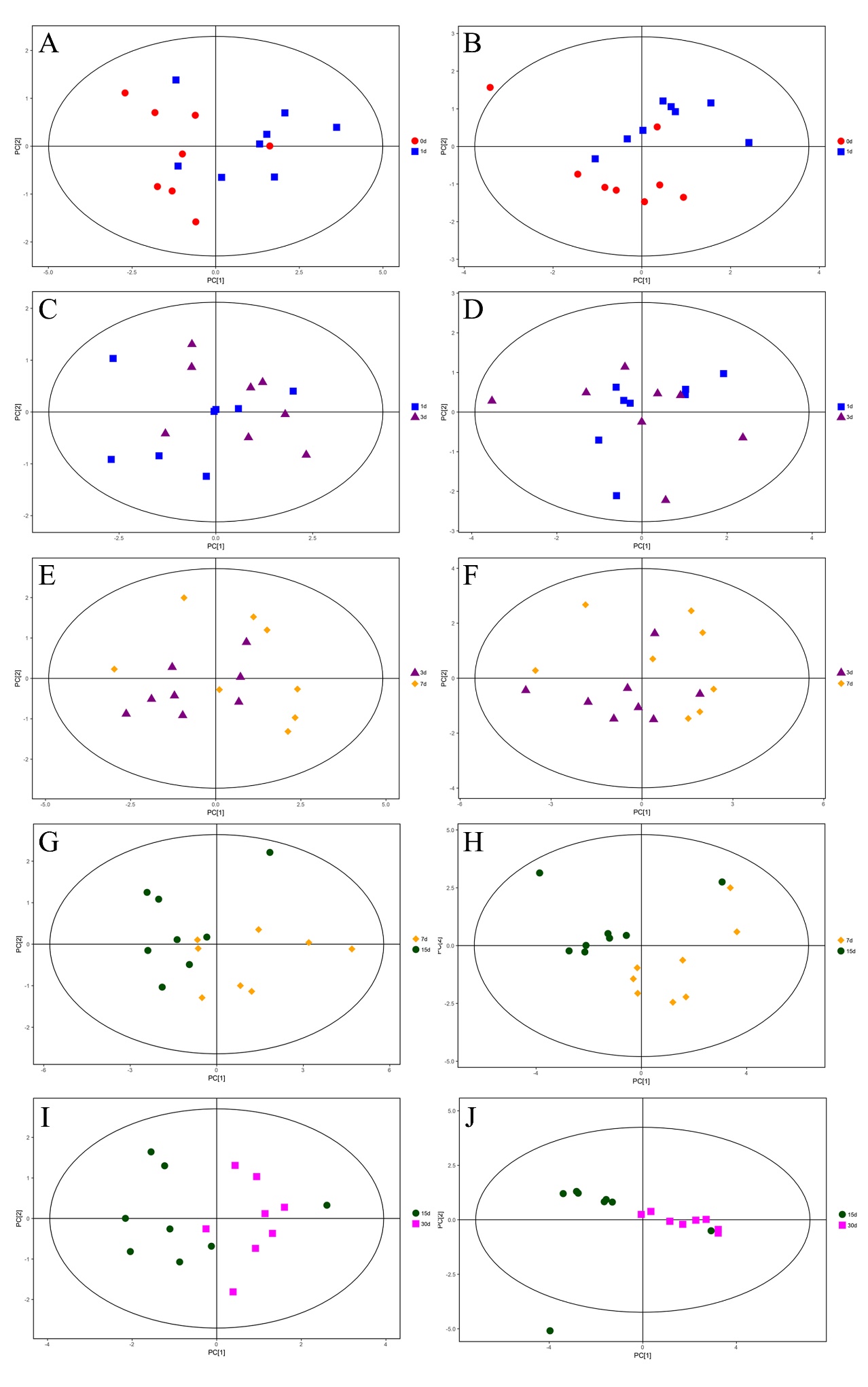


**Supplementary Fig. 2** PCA model score scatter plot for five comparison groups established for pairwise comparing different transplantation periods as follows: (A) and (B) days 0 vs. 1; (C) and (D) days 1 vs. 3; (E) and (F) days 3 vs. 7; (G) and (H) days 7 vs. 15; and (I) and (J) days 15 vs.30. A, C, E, G, and I were derived from the POS ion mode, and B, D, F, H, and J were derived from the NEG ion mode.

The PCA of are shown in Supplementary Fig. 2. The values for R2X in the PCA between days 0 and 1 were 0.66 (POS), and 0.531 (NEG). The values for R2X in the PCA between days 1 and 3 were 0.556 (POS), and 0.555 (NEG). The values for R2X in the PCA between days 3 and 7 were 0.625 (POS), and 0.689 (NEG). The values for R2X in the PCA between days 7 and 15 were 0.69 (POS), and 0.616 (NEG). The values for R2X in the PCA between days 15 and 30 were 0.589 (POS), and 0.699 (NEG).


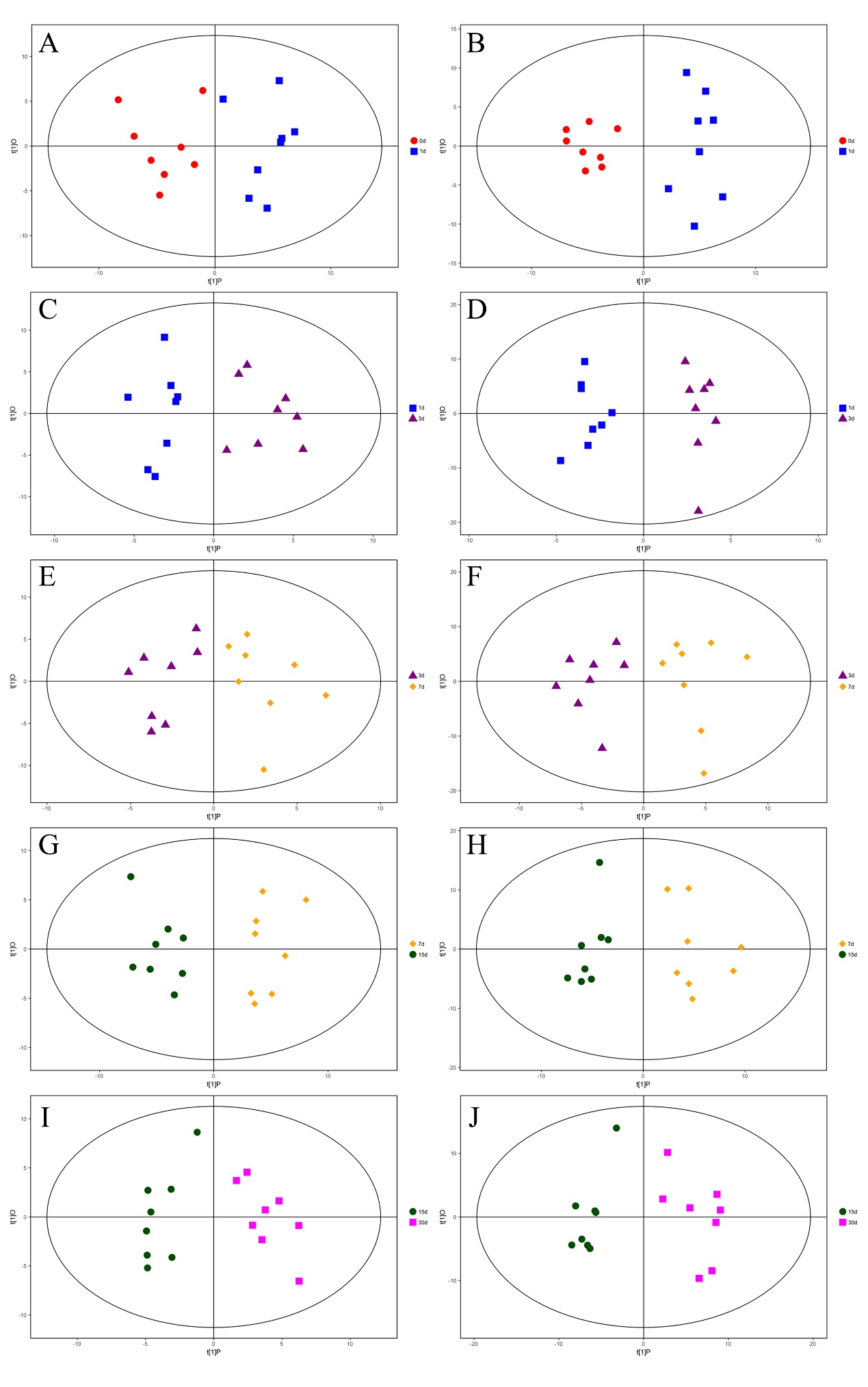


**Supplementary Fig. 3** OPLS–DA mode for five comparison groups established for pairwise comparing different transplantation periods as follows: (A) and (B) days 0 vs. 1; (C) and (D) days 1 vs. 3; (E) and (F) days 3 vs. 7; (G) and (H) days 7 vs. 15; and (I) and (J) days 15 vs.30. A, C, E, G, and I were derived from the POS ion mode, and B, D, F, H, and J were derived from the NEG ion mode.

For the two groups with additional insight, we engaged OPLS–DA to investigate divergent patterns in metabolism. The OPLS-DA results are showcased in Supplementary Fig. 3. The values for the R2X, R2Y, and Q2 using the OPLS-DA model of POS between days 0 and 1 were 0.52, 0.818, and 0.156, respectively. The values for the R2X, R2Y, and Q2 using the OPLS-DA model of NEG between days 0 and 1 were 0.379, 0.922, and 0.608, respectively. The values for the R2X, R2Y, and Q2 using the OPLS-DA model of POS between days 1 and 3 were 0.41, 0.857, and -0.0151, respectively. The values for the R2X, R2Y, and Q2 using the OPLS-DA model of NEG between days 1 and 3 were 0.415, 0.955, and 0.381, respectively. The values for the R2X, R2Y, and Q2 using the OPLS-DA model of POS between days 3 and 7 were 0.376, 0.78, and -0.167, respectively. The values for the R2X, R2Y, and Q2 using the OPLS-DA model of NEG between days 3 and 7 were 0.54, 0.838, and 0.384, respectively. The values for the R2X, R2Y, and Q2 using the OPLS-DA model of POS between days 7 and 15 were 0.474, 0.893, and 0.517, respectively. The values for the R2X, R2Y, and Q2 using the OPLS-DA model of NEG between days 7 and 15 were 0.58, 0.885, and 0.717, respectively. The values for the R2X, R2Y, and Q2 using the OPLS-DA model of POS between days 15 and 30 were 0.356, 0.883, and 0.317, respectively. The values for the R2X, R2Y, and Q2 using the OPLS-DA model of NEG between days 15 and 30 were 0.616, 0.905, and 0.738, respectively.


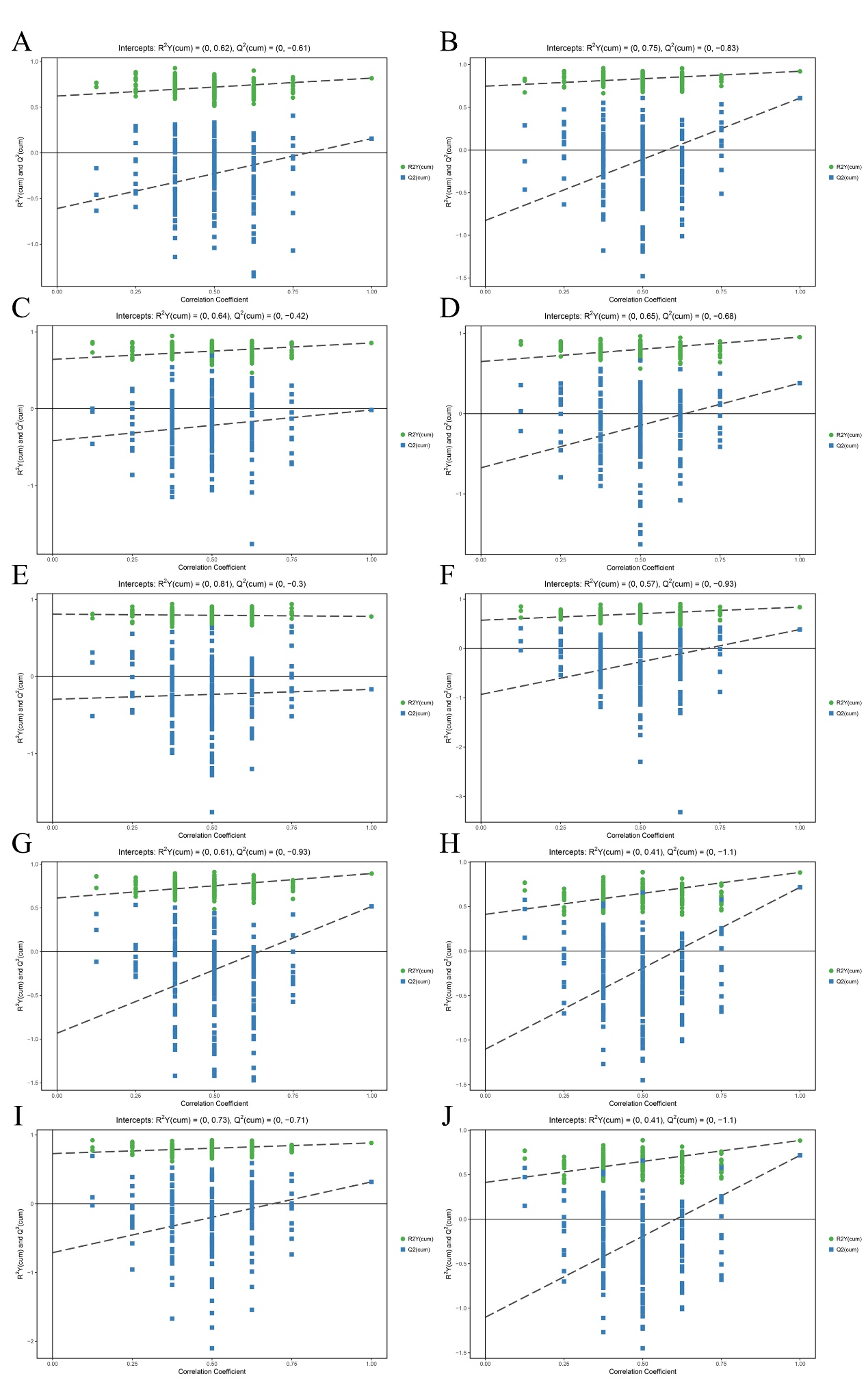


**Supplementary Fig. 4** The permutation test of OPLS–DA mode for five comparison groups established for pairwise comparing different transplantation periods as follows: (A) and (B) days 0 vs. 1; (C) and (D) days 1 vs. 3; (E) and (F) days 3 vs. 7; (G) and (H) days 7 vs. 15; and (I) and (J) days 15 vs.30. A, C, E, G, and I were derived from the POS ion mode, and B, D, F, H, and J were derived from the NEG ion mode.

The permutation test results are shown in Supplementary Fig. 4. The permutation test was applied for confirmation to remove the transition fit of the OPLS-DA model. The test results for the R2Y and Q2 intercepts between days 0 and 1 were 0.62 and −0.61 (POS), and 0.75 and −0.83 (NEG). The test results for the R2Y and Q2 intercepts between days 1 and 3 were 0.64 and −0.42 (POS), and 0.65 and −0.68 (NEG). The test results for the R2Y and Q2 intercepts between days 3 and 7 were 0.81 and −0.3 (POS), and 0.57 and −0.93 (NEG). The test results for the R2Y and Q2 intercepts between days 7 and 15 were 0.61 and −0.93 (POS), and 0.41 and −1.1 (NEG). The test results for the R2Y and Q2 intercepts between days 15 and 30 were 0.73 and −0.71 (POS), and 0.41 and −1.1 (NEG).
